# Tenascin-C expression in the lymph node pre-metastatic niche in muscle-invasive bladder cancer

**DOI:** 10.1038/s41416-021-01554-z

**Published:** 2021-09-25

**Authors:** Christopher R. Silvers, Edward M. Messing, Hiroshi Miyamoto, Yi-Fen Lee

**Affiliations:** 1 Department of Urology, University of Rochester Medical Center, Rochester, NY USA; 2 Department of Pathology & Laboratory Medicine, University of Rochester Medical Center, Rochester, NY USA; 3 Wilmot Cancer Institute, University of Rochester Medical Center, Rochester, NY USA

**Keywords:** Diagnostic markers, Bladder cancer

## Abstract

**Background:**

Markers of stromal activation at future metastatic sites may have prognostic value and may allow clinicians to identify and abolish the pre-metastatic niche to prevent metastasis. In this study, we evaluate tenascin-C as a marker of pre-metastatic niche formation in bladder cancer patient lymph nodes.

**Methods:**

Tenascin-C expression in benign lymph nodes was compared between metastatic (*n* = 20) and non-metastatic (*n* = 27) patients with muscle-invasive bladder cancer. Urinary extracellular vesicle (EV) cytokine levels were measured with an antibody array to examine potential correlation with lymph node inflammation. The ability of bladder cancer EVs to activate primary bladder fibroblasts was assessed in vitro.

**Results:**

Lymph node tenascin-C expression was elevated in metastatic patients *vs*. non-metastatic patients, and high expression was associated with worse survival. Urinary EVs contained four cytokines that were positively correlated with lymph node tenascin-C expression. Bladder cancer EVs induced tenascin-C expression in fibroblasts in an NF-κB-dependent manner.

**Conclusions:**

Tenascin-C expression in regional lymph nodes may be a good predictor of bladder cancer metastasis and an appropriate imaging target. It may be possible to interrupt pre-metastatic niche formation by targeting EV-borne tumour cytokines or by targeting tenascin-C directly.

## Background

Tumour metastasis is the main cause of cancer-related morbidity and mortality [1]. Metastases are usually only discovered after diagnosis of the original cancer using imaging assessment or histologic confirmation, and prediction of metastatic potential remains elusive—only rough predictions can be made using standard imaging, immunohistochemistry (IHC) for lymphovascular invasion (LVI), or available clinical parameters such as stage and grade of the primary tumours. The ability to make early and accurate predictions of metastases will greatly improve care for patients with cancer.

Growing evidence suggests that metastatic outgrowth is preceded by the creation of a pre-metastatic niche at future metastatic sites [2, 3]. Niches are characterised by a remodelled stromal environment that includes differentiated myofibroblasts, alterations to the extracellular matrix, activated endothelial cells and angiogenesis, and the recruitment of bone marrow-derived cells [3]. The use of sensitive stromal activation markers to detect pre-metastatic niche formation may help clinicians predict metastatic progression.

Tenascin-C is a large extracellular matrix glycoprotein that is downregulated in most healthy adult tissues and appears in a transient and highly regulated fashion during tissue remodelling [4]. In the context of inflammation and wound healing, several growth factors and cytokines have been shown to induce tenascin-C expression during myofibroblast differentiation and endothelial cell activation. In cancer, tenascin-C is highly expressed in cancer-associated fibroblasts (CAF) and endothelial cells, as well as in some cancer cells, and has been implicated in promoting survival, proliferation, migration, angiogenesis, and metastasis [5–10]. Albacete-Albacete et al. have suggested that tenascin-C might be a feature of the pre-metastatic niche [11], and as we commenced this study, we suspected that tenascin-C would be a marker of pre-metastatic stromal reprogramming in the regional lymph nodes of bladder cancer patients. In particular, the specificity of its spatial and temporal expression and its absence from most healthy tissues recommended it over another commonly used marker of fibroblast activation, alpha-smooth muscle actin (α-SMA).

Tumour-derived extracellular vesicles (EVs) are now thought to play a significant role in pre-metastatic niche formation [12]. In the primary tumour microenvironment, EVs have been shown to remodel stroma in ways that support tumour survival, proliferation, and progression [13]. Biologically active cytokines can be encapsulated within EVs or bound to the surface [14], and EVs from a variety of malignancies including bladder cancer have been shown to induce fibroblast activation in vitro by transferring encapsulated transforming growth factor-beta (TGF-β) [15, 16]. There is also mounting evidence indicating that EVs secreted by tumour cells or by activated stromal cells in the tumour microenvironment might travel through lymphatic and blood circulation and contribute to pre-metastatic niche formation. Pre-clinical studies have demonstrated this phenomenon in sentinel lymph nodes [17] and in distant organs [18–20], but we are not aware of previously published clinical evidence of EV-driven pre-metastatic niche formation in humans.

In the present work, we examine patterns of tenascin-C expression in the stroma of tumour-free lymph nodes collected from muscle-invasive bladder cancer (MIBC) patients and determine their prognostic significance. We also correlate urinary EV cargo cytokines and tenascin-C expression in the same patient’s lymph nodes. Finally, we report the effects of bladder cancer EVs on primary fibroblast tenascin-C expression in vitro. Collectively, our data suggest a mechanism of tumour EV-driven tenascin-C induction in the stroma of MIBC regional lymph nodes. While tenascin-C produced by both cancer cells [21] and stromal cells [7] has previously been shown to be a critical component of the microenvironment once metastasising cancer cells arrive, our work is the first to our knowledge to establish stromal tenascin-C induction as a feature of the pre-metastatic niche.

## Methods

### Study design

The purpose of this study was to find a useful marker of pre-metastatic niche formation in the regional lymph nodes of MIBC patients. We began with two a priori hypotheses: first, that stromal activation marker expression would be higher in the tumour-free lymph nodes of metastatic patients than in those of non-metastatic patients, and second, that high stromal activation marker expression would be associated with poor survival. Upon observing that cancer EVs were capable of upregulating activation markers in primary fibroblasts in vitro, we further hypothesised that the inflammatory cytokine content of MIBC patient urinary EVs would be positively correlated with the expression of stromal activation markers in the tumour-free lymph nodes.

Using the results of a pilot investigation of lymph node stromal marker expression, tenascin-C was prospectively chosen as the marker to be examined in patient nodes. Given the low level of tenascin-C expression in healthy lymph nodes, we anticipated an effect size >1 if the hypothesis were correct. Power analysis using *d* = 1 in a two-sample *t*-test determined that a sample size of 17 patients per group would allow the detection of a significant effect (power = 0.8). The final study group included MIBC patients with lymph node metastatic (*n* = 20) or non-metastatic disease (*n* = 27) (Supplementary Table 1). We prospectively excluded potential subjects who had a history of treatment with bacillus Calmette-Guérin (BCG). One formalin-fixed, histologically benign lymph node section from each case was selected randomly, without regard to their ipsilateral or contralateral positions relative to the primary bladder tumours or metastatic nodes. This avoided a bias in which tumour-free nodes selected for proximity to metastatic nodes would be more likely to drain the tumour and thus potentially more likely to contain evidence of a pre-metastatic niche.

For the in vitro EV studies, *TNC* and *ACTA2* were prospectively chosen as activation markers. *POSTN* expression was examined retrospectively. Each treatment group was represented by 4–8 biological replicates. For the urinary EV cytokine analysis, we included all available subjects who were BCG-naïve, who had bladder tumour >2 cm remaining at the time of cystectomy, and from whom we had a collected a sufficient amount of urinary EVs (*n* = 13). No outliers were excluded in any of the experiments.

### Immunohistochemistry

Prior to tenascin-C antigen staining, antigen retrieval was performed using proteinase K (20 µg/mL, Qiagen) for ten minutes at room temperature. For other stains, antigen retrieval was performed in ~98 °C citrate buffer (Vector Laboratories, H-3300). Tissues were incubated at 4 °C overnight with antibodies to tenascin-C (1:100, Sigma-Aldrich SAB4200782), α-SMA (1:200, Dako M0851), cytokeratins AE1/AE3 (1:300, Dako) and CAM5.2 (1:200, Dako). Staining proceeded using the standard method with avidin-biotin complex (Vector PK-6200) and the chromogen 3, 3′-diaminobenzidine (DAB) (Dako, K3466).

### Lymph node staining quantification

Stained lymph node sections were photographed using a Leica DM5000 B microscope with a ×5 objective. All photomicrographs were made with uniform illumination and exposure time and then assembled into whole-node mosaics with NIH ImageJ/Fiji [22] using a stitching plugin [23]. Lymph node total area was determined with ImageJ by selecting all pixels with grey values of 0–160 out of 255. The DAB chromogen signal of each image was extracted using the *Color Deconvolution* plugin (H DAB algorithm), and the positive DAB staining area was determined by selecting pixels with gray values 0–120 out of 255. The fraction of positive staining was calculated as DAB+ area/total tissue area. The same procedure was used to determine the DAB positive area in regions of interest (ROI) containing the most extensive staining. The procedure is illustrated in Supplementary Fig. 1.

### Cell lines

J82, TCC-SUP, and WI-38 cell lines were purchased from ATCC and maintained according to instructions. For EV collection, J82 and TCCSUP lines were cultured in CELLine bioreactor flasks (Wheaton WCL1000) using DMEM with 10% EV-depleted FBS.

### EV isolation

EVs were isolated according to a standard ultracentrifugation protocol as previously described [24, 25]. The final total protein concentration of each sample was measured using the Micro BCA assay (Thermo Scientific #23235), and samples were stored at −80 °C. We have previously documented the quality of our EV yields from both conditioned media and urine using electron microscopy, nanoparticle tracking analysis, and mass spectrometry [24, 25].

### Primary fibroblast culture

We obtained paracarcinoma bladder mucosa sections of grossly normal appearance from pN0 bladder cancer patients consented for the study. Freshly resected specimens were cut into 2 mm sections with a sterile scalpel. Sections were placed directly into tissue culture treated polystyrene flasks (Corning 430168) containing DMEM with 10% FBS and penicillin–streptomycin. Cell monolayers with fibroblast-like appearance typically emerged from the explants within two weeks. After the original sections were mechanically removed, single cells were detached using 0.25% trypsin-EDTA, transferred to new culture vessels, and maintained in DMEM containing 10% FBS and penicillin–streptomycin.

### In vitro fibroblast activation assay

The primary fibroblast cultures were used within eight passages of the initial isolation. Prior to the assays, cells were seeded in six-well culture plates, grown to >90% confluence, and then serum-starved for 72 h. For conditioned medium treatments, J82 conditioned medium was subjected to the first two steps of low-speed centrifugation in the EV isolation protocol and stored at –80 °C. When thawed, the sample was divided into two parts. The first part was set aside for use as complete conditioned medium; the second part was ultracentrifuged at 100,000 × *g* for 70 min, and the supernatant was used as EV-depleted conditioned medium. Following serum starvation, fibroblasts were grown in the conditioned media for 72 h, and then whole cell lysates were harvested.

For direct application of EVs, cells were treated with J82 EVs (*n* = 8 biological repeats) or TCCSUP EVs (*n* = 4), and healthy volunteer urinary EVs were used as EV controls (*n* = 4). DPBS treated cells were included as a control group (*n* = 6). (–)-DHMEQ (MedChem Express, HY-14645) was used at 20 µg/mL final concentration both alone (*n* = 4) and in combination with J82 EVs (*n* = 4). Equal volumes of DPBS vehicle were used in each treatment, and DMEM was added to each well for a final volume of 550 µL. In the qPCR experiments, cells were treated with an EV concentration of 100 µg/µL and harvested after 48 h; in the western blot analysis, cells were treated with 200 µg/µL EVs and harvested after 72 h.

### Total RNA extraction and quantitative real-time PCR

Total RNA was collected from cells using acid guanidinium thiocyanate-phenol-chloroform extraction and quantified using spectrophotometry (NanoDrop, Thermo Scientific). First strand cDNA was synthesised using 0.5–1 μg total RNA in a 20 μL reaction using the iScript cDNA synthesis kit instructions (Bio-Rad). cDNA levels were measured in triplicate by iQ SYBR Green (Bio-Rad), and ΔCq values were calculated using *UBC* as a reference gene. The primer sequences used are listed in Supplementary Table 2.

### Western blots

Western blots were performed with antibodies to Alix (1:300, Proteintech 12422-1-AP), TSG101 (1:1000, Santa Cruz sc-7964), PERK (1:1000, Cell Signaling Technology #5683), GAPDH (1:5000, Santa Cruz sc-32233), periostin (1:4000, Abcam ab14041), α-SMA (1:200, Dako M0851), and tenascin-C (1:200, R&D MAB2138). Alix densitometry measurements were performed with Bio-Rad Image Lab software version 5.2, and the background was subtracted using a rolling ball radius of 3 mm.

### Cytokine antibody array

Quantification of 13 cytokines in patient urinary EV specimens was performed using a custom antibody array (RayBiotech, SO2-AAH-CYT-CUST) according to the manufacturer’s instructions. Briefly, we prepared 40 µg of urinary EV protein per patient in 100 µL of DPBS. Triton X-100 was added to a final concentration of 1%, and the samples were vortexed and incubated for one hour at room temperature to permeabilize the EV membranes and allow the release of cytokines. EV samples were then diluted to 1 mL in DPBS, and protease inhibitor was included to make a 1× working concentration (Pierce, A32953). Following a 30 min block, membranes were incubated with EV samples overnight at 4 °C. Membranes were photographed with a Bio-Rad ChemiDoc XRS+, and the images were analysed with ImageJ. Background subtraction was performed on each image using a rolling ball radius of 50 pixels, and the integrated density of each dot in the blots was measured with the *MicroArray Profile* plugin. Normalisation of samples using the positive control signal intensities was completed with the RayBiotech C-Series analysis tool.

### Survival analysis

Overall survival was defined as the time from cystectomy to death from any cause. Disease-specific survival was defined as the time from cystectomy to death attributable to urothelial carcinoma. Metastasis-free survival was defined as the time from cystectomy to the first imaging of urothelial carcinoma metastasis. The survival curves were plotted by the Kaplan–Meier method and compared using the log-rank test.

### Statistical analysis

All data were analysed using the R statistical computing environment, version 3.5.3. The statistical tests used for each experiment are stated in the results section and in the relevant figure legends. The IHC quantification analysis used the non-parametric Wilcoxon rank-sum test because the data in the pN0 group were found to have a non-normal distribution using the Shapiro–Wilk test (*W* = 0.91484, *P* = 0.0297). For qPCR experiments, statistical analysis was done using ΔΔCq values, and relative expression levels were plotted using 2^−ΔΔCq^. PBS vehicle-treated samples were included by subtracting the mean PBS ΔCq from each individual PBS value. *P-*values less than 0.05 were considered statistically significant.

## Results

### Tenascin-C expression in benign lymph nodes varies between metastatic and non-metastatic patients

**Fig. 1:**
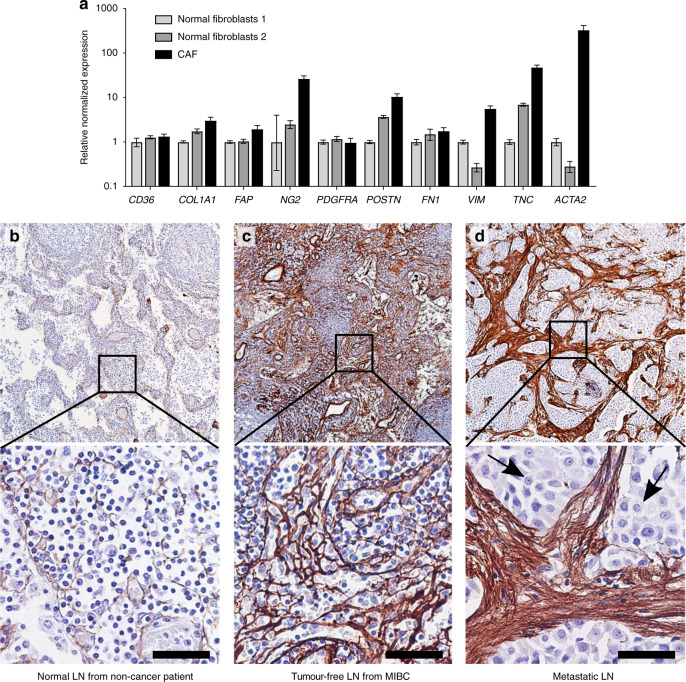
Assessment of tenascin-C expression in patient lymph nodes. **a** Expression of fibroblast activation genes in primary cultures of patient ‘normal’ bladder fibroblasts (isolated from bladder mucosa distant from the tumours) or bladder tumour-derived fibroblasts (CAF) as measured by qPCR. Error bars = SEM. **b** Tenascin-C expression in a normal lymph node (LN). This perivesical lymph node was incidentally collected from a non-cancer patient. Tenascin-C expression is indicated by DAB chromogen (brown); hematoxylin is used as a counterstain (blue). **c** A tumour-free pelvic lymph node from a patient with MIBC showing strong, diffuse tenascin-C expression. **d** Tenascin-C expression in a lymph node containing metastatic urothelial carcinoma; arrows indicate cancer cells. Scale bars = 50 µm.

To identify possible markers of lymph node metastatic niche formation, we first measured the expression of ten prospective fibroblast activation marker genes in primary CAF cells cultured from a patient tumour, comparing them to two fibroblast primary cultures derived from paracarcinoma bladder mucosa with grossly normal appearance. (Fig. 1a). *FN1* (fibronectin), a marker usually upregulated in α-SMA + CAFs, was highly expressed in both the CAF and non-CAF fibroblast cultures. Of the remaining markers, *ACTA2* (α-SMA) and *TNC* (tenascin-C) were the most highly upregulated in CAFs.

We next investigated the expression of α-SMA and tenascin-C proteins in normal perivesical lymph nodes using IHC. Expression α-SMA was widespread in the normal nodes and, therefore, not likely to be a good marker of fibroblast transition in pre-metastatic lymph nodes. Tenascin-C was minimally expressed (Fig. 1b); accordingly, we chose it as a prospective marker of stromal activation in the lymph node pre-metastatic niche.

Next, we identified a study group composed of 47 patients with a diagnosis of MIBC who underwent radical cystectomy with pelvic lymph node dissection at the University of Rochester Medical Center (Supplementary Table 1). There were 31 male and 16 female patients with a mean age of 69 years. In 19 cases tumour had invaded the muscle of the bladder wall (pathological primary tumour stage pT2); in 20 cases tumour had invaded beyond the muscle into the perivesical adipose tissue (pT3); and in 8 cases there was local spread of tumour beyond the bladder (pT4). Metastases were detected in the pelvic lymph nodes of 20 patients. Of these, 9 cases were found to have one metastatic pelvic lymph node (pathological node stage pN1), 8 cases had multiple metastatic pelvic lymph nodes (pN2), and 3 cases had positive common iliac lymph nodes (pN3). The fraction of positive nodes ranged from 1/27 (3.7%) to 14/16 (87.5%). The remaining patients in the study group (*n* = 27) had lymph nodes with no metastases (pN0).

One formalin-fixed, paraffin-embedded histologically benign node was randomly selected from each patient for evaluation of tenascin-C expression by IHC. Because it was necessary to ensure that the lymph nodes were histologically negative, an adjacent section from each node was stained with a cytokeratin antibody cocktail, and each slide was examined by a genitourinary pathologist to confirm the absence of LVI and occult micrometastases (Supplementary Fig. 2). Three of the initial 50 candidate nodes were eliminated after the discovery of cytokeratin-positive nests.

Our a priori hypothesis was that tumour-free lymph nodes from pN+ patients would have more extensive tenascin-C expression than would nodes from pN0 patients. When stained for tenascin-C, the tumour-free nodes in both groups were variable, with some showing minimal expression and others containing regions of strong, diffuse stromal staining (Fig. 1c, Supplementary Fig. 3). Histologically positive lymph nodes from four of the patients showed strong stromal tenascin-C expression, and all cancer cells appeared to be negative for tenascin-C (Fig. 1d).

**Fig. 2:**
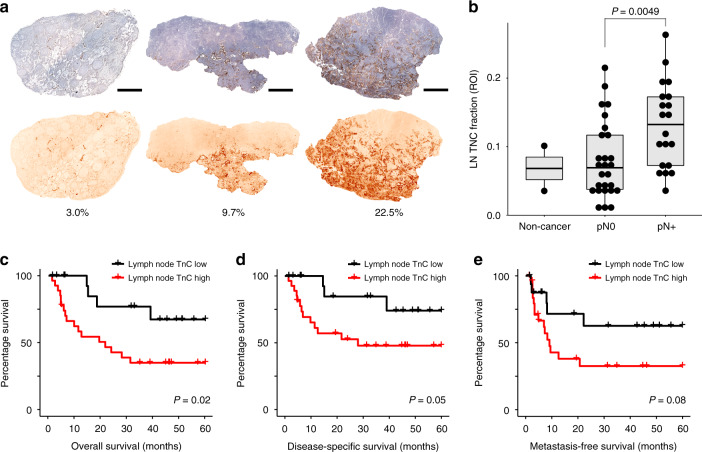
Stromal activation in MIBC regional lymph nodes. **a** Examples of varying tenascin-C expression in tumour-free lymph nodes as determined by IHC. In the top series, tenascin-C staining is indicated by DAB chromogen (brown); hematoxylin was used as a counterstain (blue). The bottom series shows the isolated tenascin-C signals following colour deconvolution with image processing software. The tenascin-C positive fraction of the total node area (minus capsule) is given as a percentage. The bottom images have enhanced contrast for the purpose of illustration. Scale bars = 1 mm. **b** Quantification of tenascin-C expression in tumour-free lymph nodes from non-metastatic (pN0, *n* = 27) or metastatic (pN+, *n* = 20) MIBC patients. Normal perivesical lymph nodes from two non-cancer patients are included for comparison. Each dot represents one node. Box plots indicate the medians and the first and third quartiles. Area fractions were determined in fields with the greatest staining intensity in each node (ROI). Comparison was made using the Wilcoxon rank-sum test. **c**–**e** Kaplan–Meier curves showing overall survival (**c**), disease-specific survival (**d**), and metastasis-free survival (**e**) in MIBC patients with high (*n* = 21) or low (*n* = 23) tenascin-C expression in tumour-free regional lymph nodes (LN TnC). Differences were calculated using the log-rank test. Survival data are given in Supplementary Table 1.

To quantify the IHC results, we selected two regions of interest (ROI) in each node that contained the most diffuse and intense staining, each field representing 2.17 mm^2^ (Supplementary Figs. 4, 5). We determined the tenascin-C positive area as a fraction of the total tissue area in each field and then took the mean (LN TnC fraction). The results showed that the pN+ group had a 1.64-fold higher mean tenascin-C expression when compared to the pN0 group (Wilcoxon rank-sum test, *P* = 0.0049; Fig. 2a, b). The difference was analysed using a non-parametric test because the pN0 group nodes were not normally distributed (Shapiro–Wilk test, *P* = 0.0297). Two alternative quantification strategies (detailed in Supplementary Fig. 6) also showed significantly more extensive tenascin-C expression in the pN+ group. We interpret this as preliminary evidence of more advanced pre-metastatic niche formation in the pN+ group.

### High tenascin-C expression in benign lymph nodes is associated with poor survival

High tenascin-C expression in primary tumour stroma predicts worse survival in a number of cancers including breast [5], colorectal [26], oesophageal squamous cell [27], and bladder [28] carcinomas. We wondered if tenascin-C expression in the stroma of draining lymph nodes would likewise predict patient outcome. Survival times for patients in the study group are included in Supplementary Table 1. The mean follow-up period was 26.4 months; 10/18 pN+ patients (55.6%) and 6/26 pN0 patients (23.1%) died from urothelial carcinoma during the follow-up period. Patients with high lymph node tenascin-C expression had worse overall survival (log-rank test, *P* = 0.02; Fig. 2c) and disease-specific survival (*P* = 0.05; Fig. 2d). Differences in metastasis-free survival were not significant (*P* = 0.08; Fig. 2e). The prognostic significance of lymph node tenascin-C expression was also calculated for the pN0 MIBC patients alone, but the number of patients was not sufficient to power a significant determination (Supplementary Fig. 9). However, the finding of the prognostic significance of tenascin-C expression in the tumour-free lymph nodes of the entire study group is further evidence of pre-metastatic niche formation in MIBC.

### Patient urinary EV cytokine levels are positively correlated with tenascin-C expression in benign lymph nodes

EVs secreted by primary bladder tumours my drain through the lymphatic vasculature and contribute to the formation of the lymph node pre-metastatic niche. Because tumour EVs also enter the bladder lumen and accumulate in the urine, the assessment of MIBC patient urinary EVs may provide some insight into the EV molecular contents that might be responsible for pre-metastatic remodelling. In the absence of direct evidence, associations between the EV cargo profile and the corresponding lymph node inflammation status might offer intriguing clues about how primary tumours influence the outlying stroma. Tumour-secreted cytokines, growth factors, and EVs are thought to be the main contributors to pre-metastatic niche formation [12]; indeed, cytokines can be secreted within EVs that protect them and facilitate their delivery to specific recipient cell types in a receptor-mediated fashion [14]. We hypothesised that the cytokine levels found in patient urinary EVs would correlate with tenascin-C expression in tumour-free patient lymph nodes. To test this, we constructed a custom antibody array to detect a panel of 13 cytokines and growth factors reported to mediate tenascin-C expression [29]. The cytokines and growth factors chosen for inclusion may in some cases induce TNC expression directly, as does TGF-β [29]; in other cases they may first activate a central regulator of inflammation such as nuclear factor κB (NF-κB) [30].

**Fig. 3:**
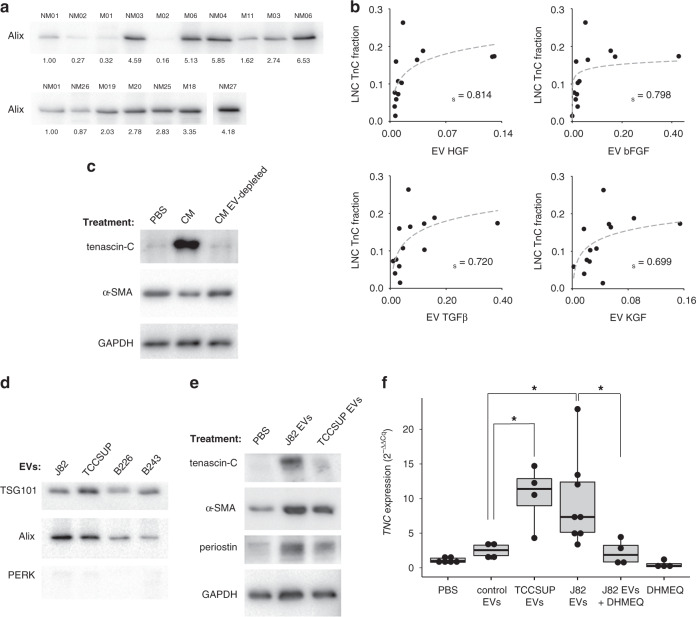
MIBC EV induction of NF-κB-dependent tenascin-C expression. **a** Western blot assessing MIBC patient urinary EVs using the exosome marker Alix. **b** Scatter plots illustrating the relationship between patient urinary EV cytokine content (arbitrary units) and tenascin-C expression in the corresponding lymph nodes (*n* = 13). Logarithmic trend lines are included. Correlations are given as Spearman’s rank correlation coefficient (*r*_*s*_). Results for 13 measured cytokines are listed in Table 1. **c** Effects of J82-conditioned medium (CM) on primary patient bladder fibroblasts in vitro shown by western blot. CM was used with and without prior EV depletion and placed on the fibroblasts for 72 h. GAPDH serves as a loading control. **d** EV isolation quality demonstrated by western blot. 10 µg of each EV sample were loaded per well. Samples B226 and B243 are patient urinary EV samples for comparison. TSG101 and Alix are EV markers. PERK, an endoplasmic reticulum resident protein, was included as a marker of contamination by intracellular material. **e** Western blot showing effects of direct bladder cancer EV treatment on primary patient bladder fibroblasts in vitro after 72 h. **f** *TNC* gene expression in primary fibroblasts treated for 48 h with bladder cancer EVs (TCCSUP and J82), non-cancer control EVs (normal volunteer urinary EVs), or PBS vehicle. J82 cancer EVs were applied with and without DHMEQ, an NF-κB inhibitor. Cells treated with DHMEQ alone are included as a control. Each dot represents one biological replicate. Box plots indicate the medians and the first and third quartiles. Differences were determined by two-sided Welch’s *t*-test.

We were able to obtain urine specimens from 22 of the MIBC patients in the initial study group at the time of cystectomy and isolate urinary EVs. Patients with bladder tumours greater than 2 cm at the time of cystectomy were included in the cytokine analysis (*n* = 13, Supplementary Table 3). Because urinary EV samples vary depending on tumour size and urine concentration, we performed a western blot to measure an exosome marker (Alix) in each sample and used this signal to normalise the cytokine results (Fig. 3a).

As seen in Fig. 3b and Table 1, four of the 13 cytokines and growth factors were positively correlated with lymph node tenascin-C expression (Spearman’s rank correlation *r*_*s*_ > 0.69, *P* < 0.05). Most notable was TGF-β, a well-documented driver of activation in a variety of stromal cell types [31]. Fibroblast activation by bladder cancer EVs containing TGF-β has previously been demonstrated in vitro [16]. Two others, basic fibroblast growth factor (bFGF) and keratinocyte growth factor (KGF/FGF7), are members of the fibroblast growth factor family. The fourth, hepatocyte growth factor (HGF), has been previously identified in EVs and implicated in pre-metastatic niche formation in animal experiments [32]. Our results are, to our knowledge, the first clinical evidence of EVs playing a role in pre-metastatic niche formation.

**Table 1:** Correlation of bladder cancer patient urinary EV cytokine levels to corresponding uninvolved lymph node tenascin C expression.

cytokine	TNC correlation (*r*_*s*_)	*P*
HGF	0.814	0.007
bFGF	0.798	0.007
TGFβ	0.720	0.026
KGF/FGF7	0.699	0.026
IL-1a	0.634	0.052
TNFα	0.498	0.146
IL-6	0.484	0.146
IGF2	0.484	0.146
PDGF-AA	0.478	0.146
MMP9	0.445	0.169
PDGF-AB	0.391	0.221
CXCL12	0.204	0.547
PDGF-BB	0.011	0.971

Urinary EVs from 13 bladder cancer patients were assessed with a cytokine antibody array. Tenascin C expression in corresponding uninvolved lymph nodes was assessed by IHC. Correlation is given as the Spearman coefficient (*r*_*s*_) and the associated probability value (*P*). *P*-values were corrected for multiple comparisons using the Benjamini–Hochberg procedure.

### Cancer EVs induce NF-κB-dependent tenascin-C expression in recipient fibroblasts in vitro

Finally, we sought to determine the effects of cancer EVs on tenascin-C expression in fibroblasts in vitro. Fibroblasts were derived from grossly normal-appearing bladder mucosa obtained from pN0 bladder cancer patients in remote locations from the index tumours, and the purity of the fibroblast isolation was confirmed using flow cytometry (Supplementary Fig. 8). When the fibroblasts were exposed to conditioned medium from J82 bladder cancer cells for 72 h, they showed strong tenascin-C protein expression (Fig. 3c); interestingly, expression of α-SMA remained unaffected. When EVs were removed from the conditioned medium by ultracentrifugation, tenascin-C was not induced (Fig. 3c), suggesting that the effect was attributable to the EV component. Next we collected EVs from both J82 and TCCSUP bladder cancer cell lines and confirmed the isolation purity with a western blot (Fig. 3d). When the cancer EVs were applied to the fibroblasts directly, the effects on tenascin-C expression were similar to those of the conditioned medium, and the fibroblast activation markers α-SMA and periostin were also upregulated (Fig. 3e).

When the fibroblasts were examined at the RNA level after 48 h of EV exposure, the tenascin-C gene was significantly upregulated. A multivariate analysis of variance was calculated for tenascin-C (*TNC*), α-SMA (*ACTA2*), and periostin (*POSTN*) across multiple treatment groups, and the analysis was significant (F(5,24) = 3.8542, *P* = 5.291 × 10^−5^). EVs from both J82 (*P* = 0.034) and TCCSUP (*P* = 0.034) induced higher *TNC* expression in the fibroblasts than did control EVs (Fig. 3f). *ACTA2* and *POSTN* were not significantly altered.

Each of the four growth factors identified in our analysis of patient urinary EVs has been shown to activate the transcription factor nuclear factor κB (NF-κB) [33–37]. NF-κB regulates the expression of several proteins characteristic of activated fibroblasts, including tenascin-C [38]. To determine whether tumour EVs upregulate tenascin-C in primary fibroblasts via the NF-κB pathway, we included an NF-κB inhibitor, dehydroxymethylepoxyquinomicin (DHMEQ) [39], in combination with J82 EV treatment. NF-κB inhibition abolished the EV effect on *TNC* upregulation (*P* = 0.015; Fig. 3f). The use of DHMEQ alone produced an insignificant downregulation of *TNC* expression compared to the vehicle control. These data suggest that the EV-mediated effects on tenascin-C expression in fibroblasts in vitro are dependent on the NF-κB pathway, and they further strengthen the connection between patient urinary EV cytokine content and the corresponding lymph node tenascin-C expression.

## Discussion

In the present study, we have identified tenascin-C as a specific, highly-expressed feature of the lymph node pre-metastatic niche in MIBC. The work is limited by small sample size, and the observations require validation in a larger series prior to the use of tenascin-C as a target in the clinic. In particular, it would be important to determine the prognostic significance of lymph node tenascin-C expression strictly within pN0 MIBC patients. Additionally, sampling error could be reduced by the assessment of multiple lymph nodes per patient, and the patterns of pre-metastatic niche formation in multiple tumour-draining nodes could be explored in greater depth. To confirm the ability of individual EV cytokines to induce tenascin-C in fibroblasts, loss-of-function experiments would be required. Longer treatments of fibroblasts with cancer EVs might also recapitulate other interesting features of the CAF phenotype.

The strength of this work lies in its use of human samples to demonstrate pre-metastatic niche formation. The question is addressed from multiple angles in terms of differential expression (pN+ vs. pN0), patient survival, and urinary EV cytokine correlations, each of which reflects human biology. Clinical data are often more relevant than data derived from mouse models, which offer poor simulations of human conditions such as inflammation. For instance, Seok et al. reported that the transcriptional response to acute inflammatory stress in mouse models weakly resembled the condition in humans, concluding that clinical data should be prioritised when seeking a rationale for therapeutic targets [40]. Our study provides clinical data supporting the role of inflammation in the pre-metastatic niche and the possible involvement of EV-borne cytokines.

Future work arising from this study may continue to examine the role of cytokines in inducing stromal remodelling at pre-metastatic sites, but alternative mechanisms should not be neglected. For example, tumour-derived EVs are known to contain various non-coding RNAs (ncRNA) that regulate numerous cellular functions that favour cancer growth and progression [41]. MiR-198 in colon cancer stroma [42] and LncRNA AK033210 in lung tissues [43] are found to be associated with TNC levels. Thus, it might be of interest to determine if urinary EVs from MIBC patients contain ncRNAs that can regulate TNC.

Additionally, EVs are emerging as important carriers of extracellular matrix components [11], and in particular it has been suggested that most tenascin-C deposition into the extracellular matrix is via caveolin-1-positive exosomes [44]. It would be interesting to determine whether tumour or stromal EVs deliver tenascin-C from the tumour region to pre-metastatic sites and thus contribute to extracellular matrix remodelling or other aspects of pre-metastatic niche conditioning.

Most of the data supporting the pre-metastatic niche hypothesis have been obtained using in vitro assays and murine models[3]. In humans, regional lymph node dissection in the treatment of various cancers provides an opportunity to examine potentially pre-metastatic tissue, and clinical evidence supporting the hypothesis has been accumulating through the use of various strategies. In a study of oral squamous cell carcinoma patients, Chung et al. used nuclear imaging to distinguish between sentinel and non-sentinel lymph nodes, and tumour-free sentinel nodes were found to have higher lymphatic vessel density than non-sentinel nodes [45]. Otto et al. examined the RNA transcriptomes in tumour-free nodes collected from pN1 and pN0 oesophageal cancer patients and detected distinct molecular profiles [46]. In a study of MIBC patients, Pal et al. recently reported that increased neutrophil infiltration in benign nodes was associated with poor survival [47]. Our work describing the prognostic significance of tenascin-C in benign lymph nodes and its differential expression in nodes from pN+ and pN0 MIBC patients contributes to these efforts.

Clinical prediction of metastatic potential will greatly advance patient care. Markers could include altered tumour secretory profiles (including altered blood or urinary EV profiles) or stromal activation at pre-metastatic sites. Near-term applications could include sensitive molecular probes used during regional lymph node dissection to identify metastatic lymph nodes as well as nodes with pre-metastatic stromal alterations. Tenascin-C is a highly specific stromal activation marker, and in vivo detection of tenascin-C has been progressing in various fields. Anti-tenascin-C antibodies conjugated to iron oxide nanoparticles have been used as magnetic resonance imaging probes to detect myocardial infarction [48] and atherosclerosis [49] in mice. An ^111^In-labelled anti-tenascin-C antibody has been used to detect myocardial infarction in cynomolgus monkey hearts [50]. Recently, anti-tenascin-C single domain nanobodies have been developed that may also prove useful in a variety of imaging or compound-delivery applications [51], and other emerging techniques use radiolabeled or fluorescence-labelled tenascin-C aptamers for tumour detection [52, 53].

Due to its large size and high level of expression, tenascin-C may also be useful drug-delivery target. Tenascin-C has already been targeted by a recombinant antibody–cytokine fusion protein for delivery of IL-2 into tumours [54], and it may be that a similar approach could be used to deliver compounds to the pre-metastatic niche. Anti-tenascin-C nanobodies are also potentially attractive in this application given their high specificity and small size [51]. Tenascin-C has also been used as a target for radioimmunotherapy [55], but the value of attacking stroma directly in this manner is disputed, as radiation may increase inflammation and stromal activation [56]. Another consideration when targeting stromal cell types is that it usually requires a surface marker, and tenascin-C is secreted into the extracellular matrix. Targeting the secreted protein itself for degradation may not be straightforward either. Proteolytic cleavage of full length tenascin-C results in the loss of some functions and the appearance of others as cryptic binding sites are revealed [57].

In conclusion, this work presents clinical evidence of the lymph node pre-metastatic niche and establishes tenascin-C as a feature of the niche prior to the arrival of tumour cells. The finding of an association between urinary EVs and inflammation in regional lymph node stroma provides clinical evidence of EV-mediated pre-metastatic niche formation. Finally, our data suggest a mechanism in which tumour EV cytokines promote niche formation and offer tractable targets for therapeutic intervention that may allow the prevention of metastases.

## Supplementary information

aj checklist

Supplementary information

## Data Availability

Most of the data associated with this study are available in the main text or the Supplementary materials. Raw data such as IHC photographs (Figs. 1 and 2, Supplementary Figs. 2–5), cytokine array photographs (Fig. 3b), western blots (Fig. 3) and qPCR data (Fig. 3f) will be made available upon request.
