# Supplementary material for: Tenascin-C expression in the lymph node pre-metastatic niche in muscle-invasive bladder cancer
Source: Br J Cancer. 2021 Sep 25;125(10):1399–407. doi: 10.1038/s41416-021-01554-z (PMC8575937; doi:10.1038/s41416-021-01554-z)
Supplement: Supplementary file 2 — Supplementary information [file 41416_2021_1554_MOESM2_ESM.pdf]

Supplementary Information

Contents

Supplementary Fig. 1 2

Supplementary Fig. 2 3

Supplementary Fig. 3 4

Supplementary Fig. 4 5

Supplementary Fig. 5 6

Supplementary Fig. 6 7

Supplementary Fig. 7 8

Supplementary Fig. 8 9

Supplementary Table 1 10

Supplementary Table 2 11

Supplementary Table 3 12

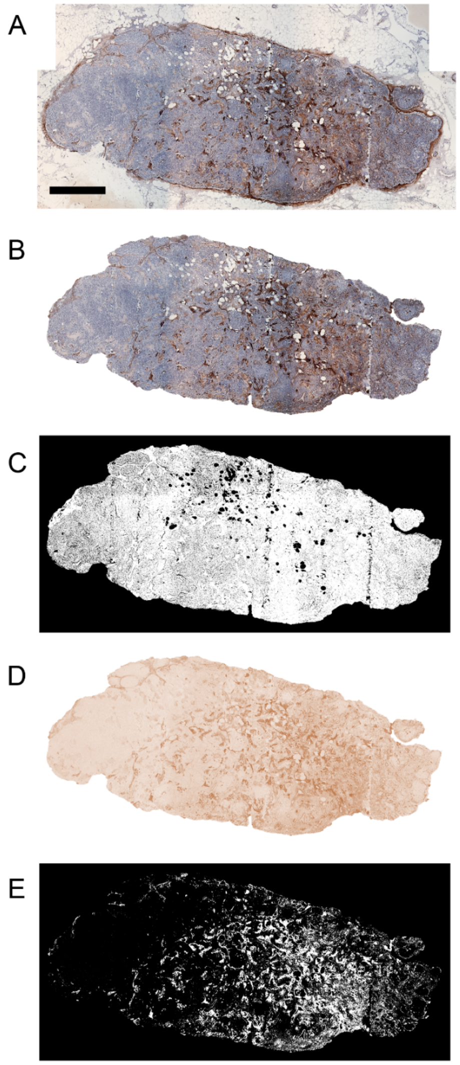

Supplementary Fig. 1: Area quantification of tenascin-C expression using ImageJ analysis software. **A** Mosaic photomicrograph of a whole lymph node section stained for tenascin-C. Scale bar = 1 mm. **B** Node area isolated and capsule removed. **C** Selection of the total node tissue area (white). **D** Isolation of the DAB chromogen signal from the color IHC image by color deconvolution. **E** Selection of DAB-positive area (white) above a specified threshold. The tenascin-C-positive area as a fraction of the total node area (C) can then be determined.

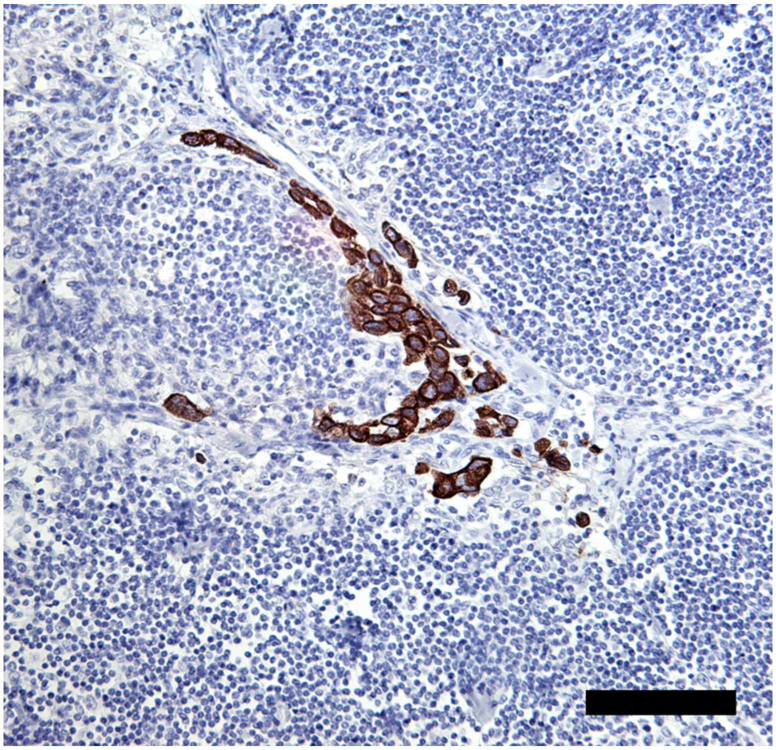

Supplementary Fig. 2: Assessment of lymph nodes for occult micrometastases. IHC was performed on serial lymph node sections using a cocktail of antibodies against cytokeratins AE1/AE3 and CAM5.2. DAB staining (brown) indicates the presence of cancer cells.

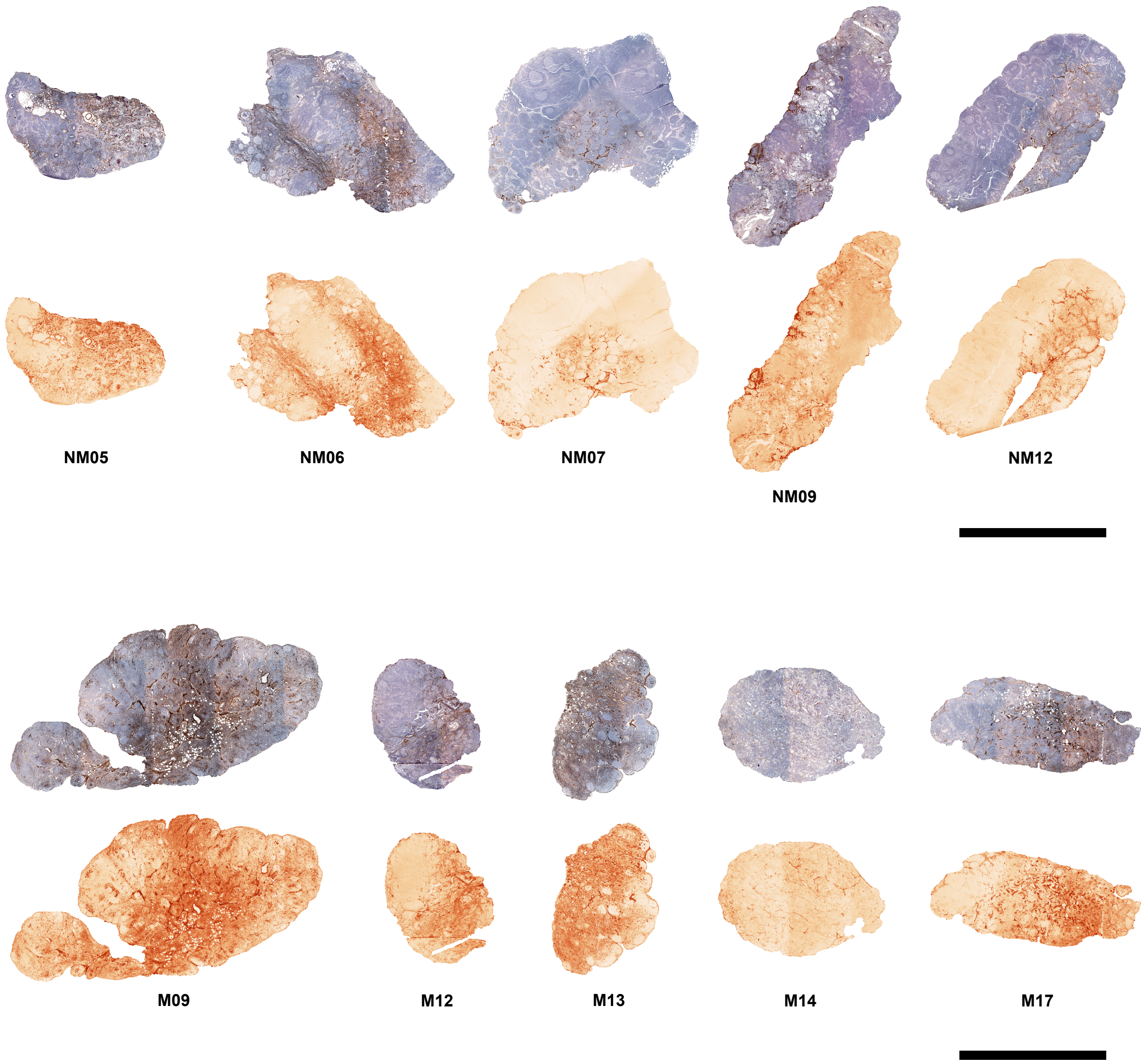

Supplementary Fig. 3. Representative tenascin-C staining in whole-mount lymph nodes. Five benign nodes are presented for non-metastatic (NM, top rows) and metastatic (M, bottom rows) bladder cancer patients. The DAB chromogen signal (brown) was isolated by color deconvolution and indicates tenascin-C expression. Scale bars = 5 mm

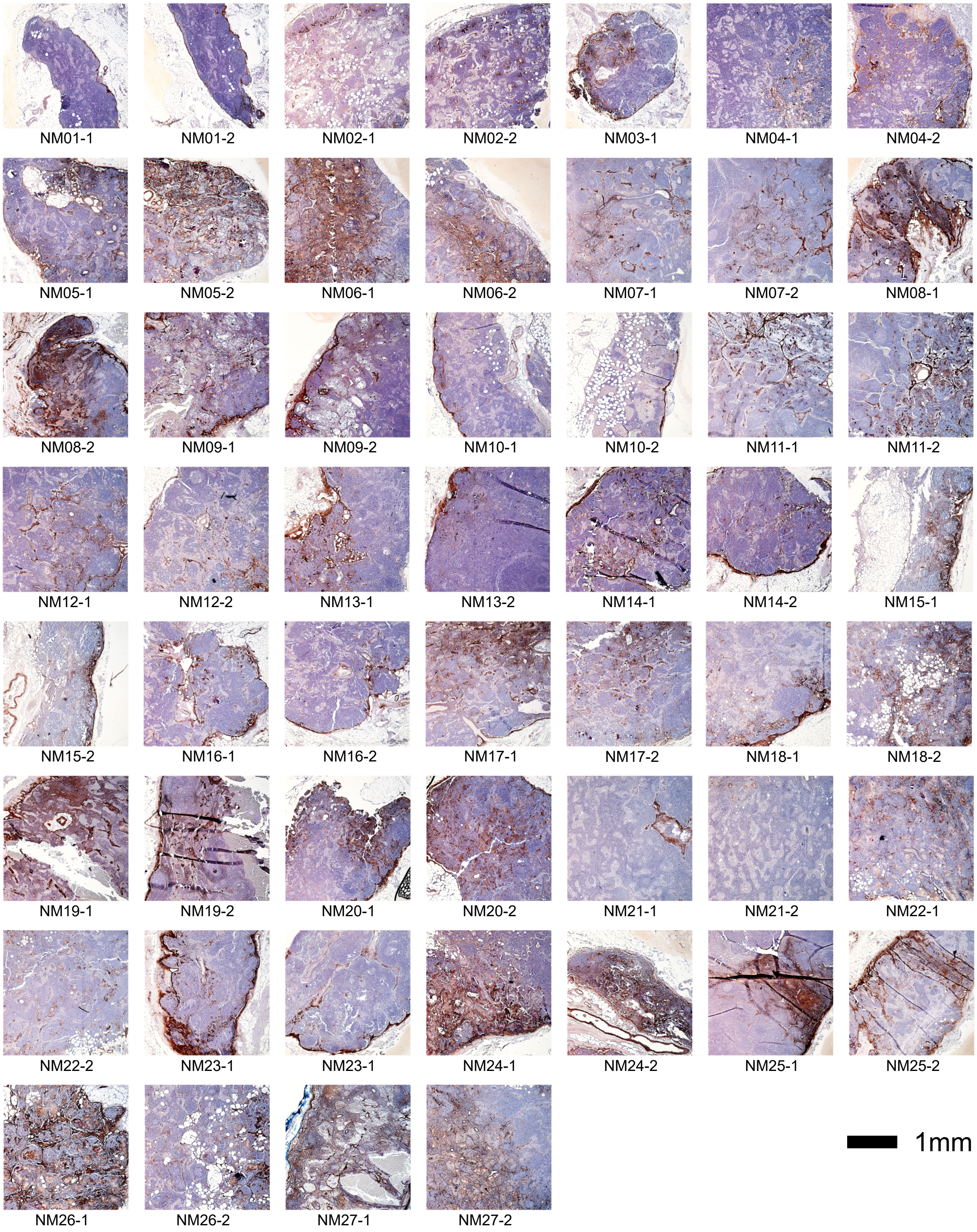

Supplementary Fig. 4. Benign lymph nodes from non-metastatic bladder cancer patients, 50× fields. One node was randomly selected from each case. Two fields were selected from each node except NM03. Nodes from metastatic patients in the study group are shown in Supplementary Fig. 5. Patient data are given in Supplementary Table 1. Scale bar = 1 mm

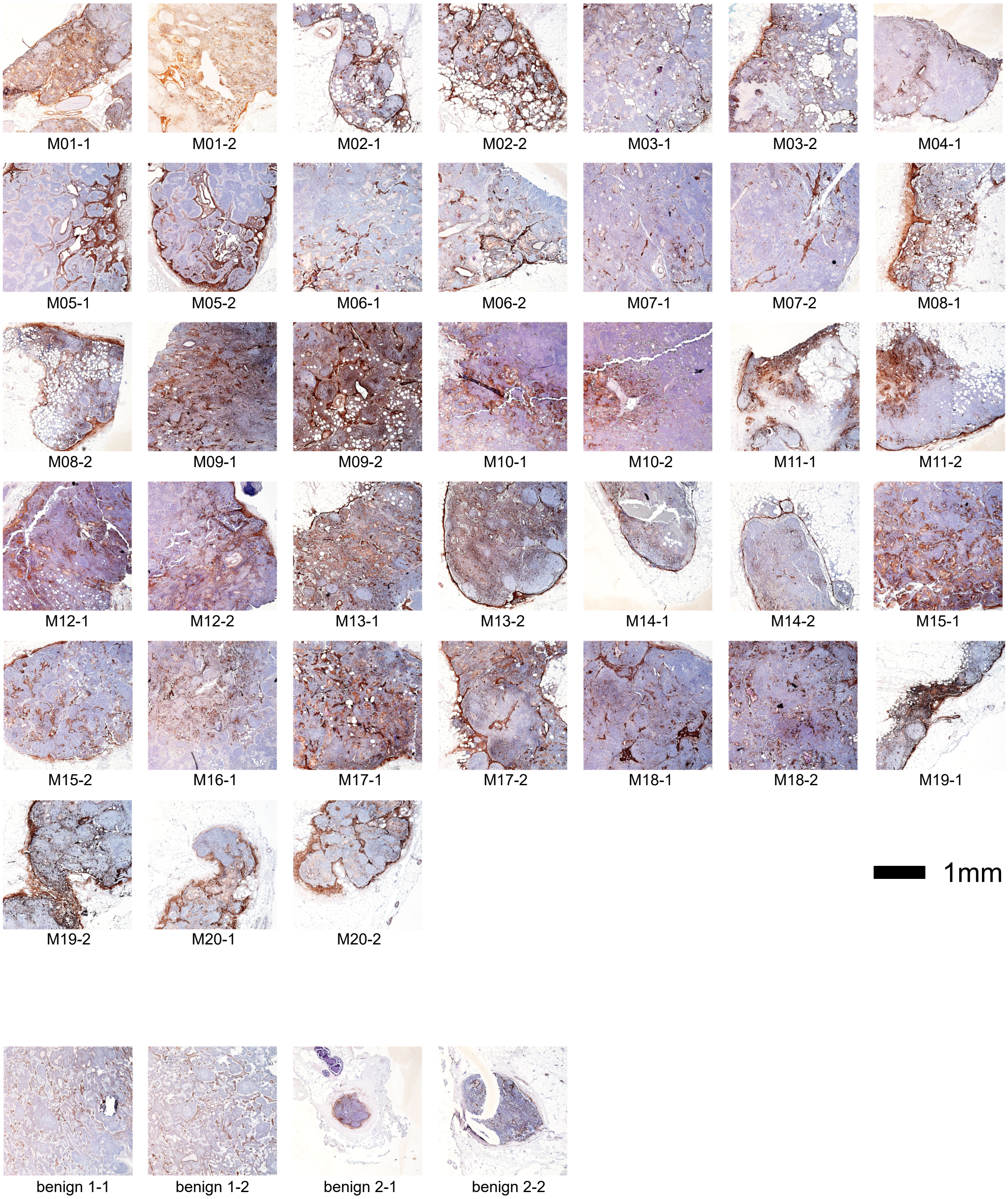

Supplementary Fig. 5. Benign lymph nodes from metastatic bladder cancer patients, 50× fields. Control nodes from two patients who did not have cancer are included at botttom. Scale bar = 1 mm

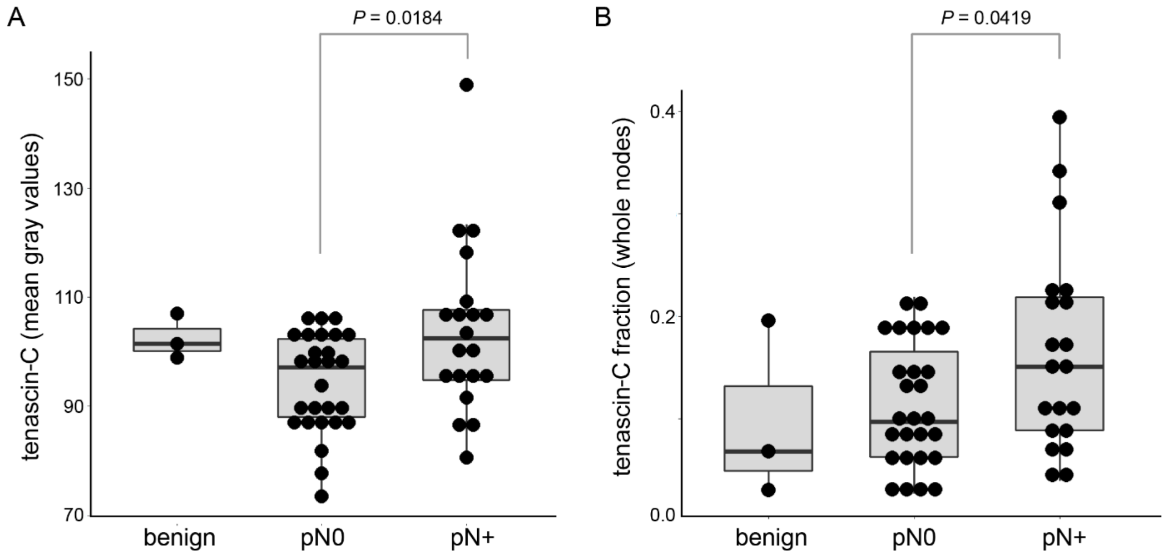

Supplementary Fig. 6: Alternative methods of quantifying IHC tenascin-C staining in uninvolved patient lymph nodes. **A** The mean gray value of all pixels within the lymph node tissue area calculated for each whole-node DAB deconvolution image. **B** The DAB-positive area fraction calculated for each whole node DAB deconvolution image. Each dot represents one node. Box plots indicate the medians and the first and third quartiles. Differences were determined using Welch's *t*-test.

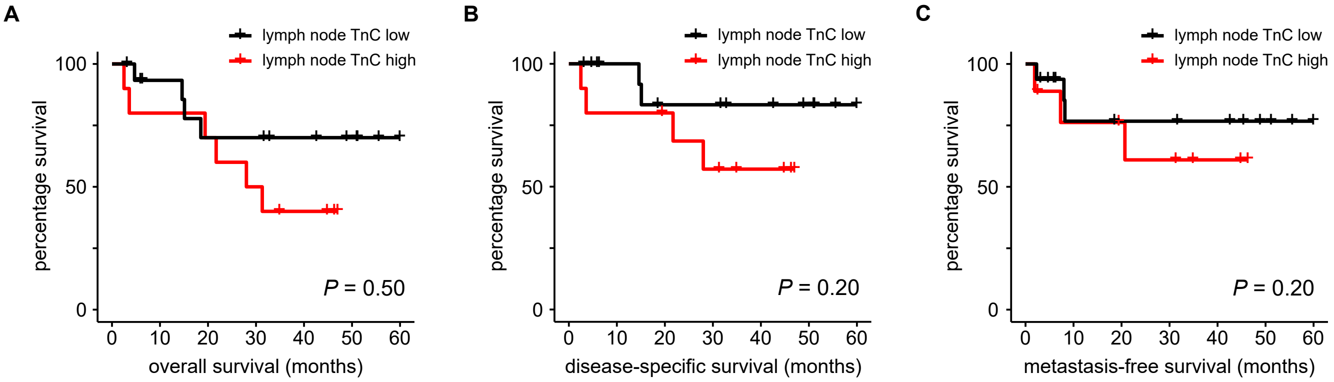

Supplementary Fig. 7: Non-metastatic MIBC patient survival. Kaplan-Meier curves showing overall survival (A), disease-specific survival (B), and metastasis-free survival (C) in non-metastatic MIBC patients with high ($n = 10$) or low ($n = 16$) tenascin-C expression in benign regional lymph nodes (LN TnC). Differences were calculated using the log-rank test. Survival data are given in Supplementary Table 1.

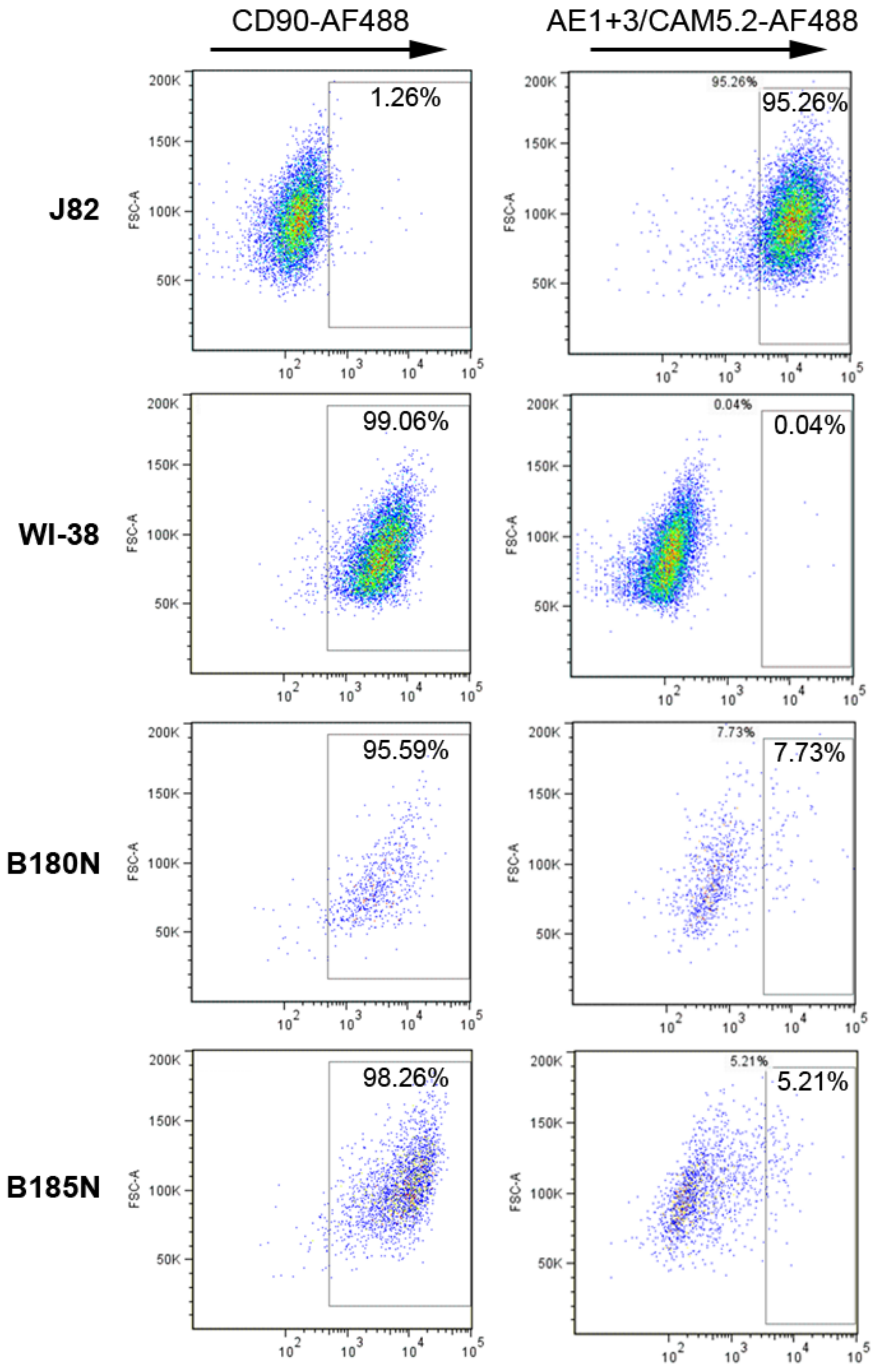

Supplementary Fig. 8: Flow cytometric evaluation of human bladder fibroblast primary cultures. A stromal marker (CD90) and an epithelial marker cocktail (AE1+3 and CAM5.2) were used to label cells from an epithelial cell line (J82), a lung fibroblast cell line (WI-38), and two primary cultures derived from paracarcinoma bladder mucosa (B180N and B185N).

ID	age	sex	pT	pN	lymph node TnC	OS (months)	DSS (months)	MFS (months)
NM01	77.1	M	2a	0	0.0149	31.6*	31.6*	31.6*
NM02	77.4	F	2a	0	0.0447	3.1*	3.1*	3.1*
NM03	65.3	F	2a	0	0.0769	60.0*	60.0*	60.0*
NM04	80.2	F	2b	0	0.0399	60.0*	60.0*	60.0*
NM05	55.8	F	3a	0	0.1128	46.3*	46.3*	46.3*
NM06	65.6	M	3a	0	0.1596	44.8*	44.8*	44.8*
NM07	77.3	M	4a	0	0.0357	55.6*	55.6*	55.6*
NM08	72.3	F	3b	0	0.1209	21.7	21.7	1.9
NM09	61.6	M	2b	0	0.0806	28.0	28.0	20.7
NM10	37.2	M	3b	0	0.0119	15.1	15.1	8.2
NM11	55.0	F	2b	0	0.0602	48.9*	48.9*	48.9*
NM12	63.1	M	3a	0	0.0412	14.6	14.6	8.0
NM13	66.9	F	2a	0	0.0458	5.9*	5.9*	5.9*
NM14	80.1	M	3a	0	0.0693	51.0*	51.0*	45.4*
NM15	78.7	M	2a	0	0.0708	4.7	4.7*	4.7*
NM16	87.1	F	3a	0	0.0321	51.2*	51.2*	51.2*
NM17	69.9	M	2b	0	0.0868	31.3	31.3*	31.3*
NM18	60.0	F	2b	0	0.0591	42.6*	42.6*	42.6*
NM19	62.1	M	3a	0	0.1458	47.0*	47.0*	7.3
NM20	70.9	F	2a	0	0.1275	--	--	--
NM21	61.2	M	2a	0	0.0076	32.8*	32.8*	2.3
NM22	79.1	F	2a	0	0.0342	6.3*	6.3*	6.3*
NM23	65.7	M	2b	0	0.0329	18.5	18.5*	18.5*
NM24	83.6	M	4a	0	0.2151	19.4	19.4*	19.4*
NM25	77.8	M	3b	0	0.0792	3.6	3.6	--
NM26	71.8	M	3a	0	0.1881	2.5	2.5	2.5*
NM27	62.3	F	3b	0	0.1641	34.9*	34.9*	34.9*
M01	63.9	F	2a	1	0.1736	12.6	12.6	9.1
M02	76.1	M	3b	2	0.1470	5.0*	5.0*	5.0*
M03	74.3	M	3a	1	0.0727	5.8	5.8	3.1
M04	70.4	M	4a	2	0.0597	--	--	--
M05	54.7	M	4b	2	0.0723	--	--	--
M06	72.7	M	2a	1	0.0589	39.0	39.0	22.2
M07	80.4	M	3b	3	0.0358	1.5*	1.5*	1.5*
M08	70.5	M	3a	1	0.1624	11.4	11.4	2.7
M09	69.0	M	2b	2	0.1930	60.0*	60.0*	60.0*
M10	57.2	F	2b	3	0.1187	4.5	4.5	3.4
M11	63.9	M	2	1	0.1028	57.1*	57.1*	9.5
M12	68.4	M	3b	1	0.1580	1.4	1.4	1.4*
M13	73.3	F	3a	1	0.2231	46.0*	46.0*	8.3
M14	46.4	F	4a	1	0.0638	45.3*	45.3*	2.0
M15	69.6	M	4a	2	0.1460	4.7	4.7	2.6
M16	60.3	M	4a	1	0.1001	6.4	6.4	3.4
M17	70.8	M	3a	3	0.1962	9.8	9.8	7.0
M18	69.1	M	3b	2	0.1073	3.6	3.6	--
M19	71.4	M	3a	2	0.2633	2.5	2.5	2.5*
M20	79.2	M	3b	2	0.1724	34.9*	34.9*	34.9*

Supplementary Table 1: MIBC patients evaluated for tenascin-C expression in benign lymph nodes. Pathological tumor staging (pT) indicated the presence of muscle-invasive tumor ($\geq$pT2) at the time of cystectomy. Resected lymph nodes were examined for the presence of metastatic urothelial carcinoma (pN-stage). Tenascin-C expression in benign lymph nodes is given as the positive-staining fraction of the total tissue area in the images. Survival times are given for overall survival (OS), disease-specific survival (DSS), and metastasis-free survival (MFS). Asterisks (*) indicate censored data.

gene	NCBI ID	size	name	primer sequence (5' - 3')
ACTA2	59	109	h SMA F	GTGTTGCCCCTGAAGAGCAT
			h SMA R	GCTGGGACATTGAAAGTCTCA
CD36	948	92	h CD36 F	GGCTGTGACCGGAACTGTG
			h CD36 R	AGGTCTCCAACTGGCATTAGAA
COL1A1	1277	119	h COL1A1 F	GTGCGATGACGTGATCTGTGA
			h COL1A1 R	CGGTGGTTTCTTGGTCGGT
FAP	2191	103	h FAP-a F	TGAACGAGTATGTTTGCAGTGG
			h FAP-a R	GGTCTTTGGACAATCCCATGT
FN1	2335	146	h fibronectin F	CCAGGCACTGACTACAAGAT
			h fibronectin R	CATGATACCAGCAAGGACTT
NG2	1464	202	h NG2 F	CTTTGACCCTGACTATGTTGGC
			h NG2 R	TGCAGGCGTCCAGAGTAGA
PDGFRA	5156	119	h PDGFRA F	TTGAAGGCAGGCACATTTACA
			h PDGFRA R	GCGACAAGGTATAATGGCAGAAT
POSTN	10631	138	h POSTN F	CTCATAGTCGTATCAGGGGTCG
			h POSTN R	ACACAGTCGTTTTCTGTCCAC
TNC	3371	131	h TNC F	TCCCAGTGTTCGGTGGATCT
			h TNC R	TTGATGCGATGTGTGAAGACA
UBC	7316	117	h UBC F	CTGGAAGATGGTCGTACCCTG
			h UBC R	GGTCTTGCCAGTGAGTGTCT
VIM	7431	98	h vimentin F	AGTCCACTGAGTACCGGAGAC
			h vimentin R	CATTTCACGCATCTGGCGTTC

Supplementary Table 2: Sequences of primers used in the qPCR experiments. Size indicates the length of the amplicon produced by the given pair of primers.

patient ID	NM01	NM03	NM04	NM06	NM26	NM27	M01	M03	M06	M11	M18	M19	M20
age	77.1	65.3	80.2	65.6	71.8	62.3	63.9	74.3	72.7	63.9	69.1	71.4	79.2
sex	M	F	F	M	M	F	F	M	M	M	M	M	M
pT	2a	2a	2b	3a	3a	3b	2a	3a	2a	2	3b	3a	3b
pN	0	0	0	0	0	0	1	1	1	1	2	2	2
LN TnC	0.0149	0.0769	0.0399	0.1596	0.1881	0.1641	0.1736	0.0727	0.0589	0.1028	0.1073	0.2633	0.1724
WB Alix	1.00	4.59	5.85	6.53	0.87	4.18	0.32	2.74	5.13	1.62	3.35	2.03	2.78
MMP9	0.293	0.035	0.356	0.303	1.837	0.135	21.264	1.396	0.027	0.200	0.209	0.309	3.338
IL-1a	0.000	0.002	0.009	0.002	0.037	0.035	0.261	0.000	0.010	0.041	0.008	0.012	0.023
IL-6	0.000	0.000	0.005	0.000	0.035	0.035	0.000	0.000	0.010	0.013	0.004	0.020	0.045
CXCL12	0.000	0.000	0.002	0.001	0.000	0.049	0.000	0.000	0.009	0.045	0.005	0.017	0.039
PDGF-AA	0.167	0.031	0.122	0.053	0.273	0.070	0.713	0.056	0.028	0.172	0.068	0.136	0.481
PDGF-AB	0.027	0.003	0.032	0.017	0.000	0.047	0.426	0.000	0.013	0.044	0.028	0.046	0.061
PDGF-BB	0.047	0.000	0.105	0.010	0.098	0.045	0.000	0.000	0.008	0.023	0.025	0.018	0.051
bFGF	0.000	0.008	0.022	0.016	0.154	0.063	0.428	0.019	0.014	0.022	0.029	0.053	0.172
HGF	0.007	0.006	0.006	0.008	0.041	0.034	0.131	0.010	0.008	0.015	0.010	0.016	0.128
IGF2	0.136	0.000	0.030	0.035	0.192	0.064	0.584	0.031	0.044	0.109	0.040	0.082	0.126
TNFa	0.119	0.020	0.032	0.012	0.230	0.069	0.242	0.037	0.027	0.119	0.043	0.077	0.168
TGFb	0.035	0.021	0.017	0.033	0.159	0.072	0.384	0.011	0.032	0.123	0.046	0.065	0.122
KGF	0.044	0.021	0.017	0.017	0.088	0.055	0.154	0.025	0.002	0.026	0.035	0.045	0.053

Supplementary Table 3: Cytokine antibody array data. Thirteen patients from Supplementary Table 1 were selected for measurement of urinary EV cytokine levels using an antibody array. The cytokine levels given are normalized using the Alix densitometry value as determined by Western blot (Fig. 3a). The tenascin-C positive area fraction in an uninvolved regional lymph node (LN TnC) was previously determined.
